# Education and training for health professionals on disability inclusion: a scoping review

**DOI:** 10.1186/s12909-026-09492-1

**Published:** 2026-06-20

**Authors:** Lea Gölz, Julius Rosenhan, Kaloyan Kamenov, Birgit Prodinger

**Affiliations:** 1 Inclusive Health Care, Faculty of Medicine, University of Augsburg, Augsburg, Germany; 2 Disability Programme, Crosscutting and Integration Unit, Department for Noncommunicable diseases and Mental Health, World Health Organization, Geneva, Switzerland; 3 Policy and Global Advocacy, Sightsavers, Dar es Salaam, Tanzania

**Keywords:** Competency-based education, Human rights, Disability inclusion; health equity; medical education, Health professional education

## Abstract

**Background:**

Persons with disabilities continue to experience profound and avoidable health inequities. Health professionals play a key role in addressing these inequities yet often lack the competencies and preparedness to provide equitable and rights-based care. To advance disability inclusion within health professions education, clarity is needed on which themes are currently represented in existing education and trainings.

The objectives of this scoping review are 1) to describe how disability-related content has been incorporated into education and training for health professionals, and 2) to identify the key themes addressed in the existing literature.

**Methods:**

A scoping review was conducted following the methodological framework by Arksey and O’Malley and the PRISMA-ScR reporting guidelines. Literature published between January 2011 and August 2024 was searched in MEDLINE, Web of Science, Google, Google Scholar, and relevant organisational websites. Both academic and grey literature were included. Data were charted according to study characteristics, educational scope, understanding of disability, involvement of persons with disabilities, teaching format, and targeted health professionals. An inductive thematic analysis identified key themes addressed in education and training.

**Results:**

A total of 172 publications met the inclusion criteria. The majority originated from the Region of the Americas and the European Region and were locally or nationally implemented initiatives. Nineteen distinct themes were identified. These themes ranged from themes such as conceptual understanding of disability and the perspectives of persons with disabilities to specific themes such as discrimination and violence or costs and time management. Most training interventions were small sessions or modules rather than longitudinal curricula, and few used validated tools to evaluate learning outcomes.

**Conclusions:**

Disability-related education and training for health professionals are expanding but remain fragmented and context-specific. There is an urgent need for globally aligned competency standards to guide curriculum development, for longitudinal integration of disability inclusion throughout training, and for the use of reliable evaluation frameworks. Strengthening competency- and human rights-based education is essential to prepare health professionals to deliver equitable, inclusive, and high-quality care for persons with disabilities.

**Trial registration:**

Clinical trial number not applicable.

**Supplementary Information:**

The online version contains supplementary material available at 10.1186/s12909-026-09492-1.

## Background

Globally, it is estimated that 1.3 billion people, representing approximately 16% of the global population, have a disability [1]. Despite having the right to the enjoyment of the highest attainable standard of health as any other person [1–3], persons with disabilities experience a range of health inequities – higher rates of mortality, poorer health and functioning compared to the rest of the population [1, 4]. These health inequities are often driven by unfair and avoidable factors in the health system, in which the health workforce plays a pivotal role. Health professionals often lack the competencies, confidence, skills and preparedness to provide equitable, accessible, and rights-based care for persons with disabilities. This often leads to discriminatory attitudes, implicit biases, and stigmatizing behaviours towards persons with disabilities [1, 5–7], which constitute barriers in communication, access, and recognition of their rights within health systems [1, 4–10].

To address this gap, it is crucial that health workers receive education and training on disability inclusion. Disability inclusion goes beyond clinical competencies related to specific impairments; it refers to understanding and addressing the social, environmental, and attitudinal barriers that limit the participation of persons with disabilities in health care. It promotes the meaningful inclusion of persons with disabilities in all their diversity and the recognition of their rights in line with the United Nations Convention on the Rights of Persons with Disabilities (UN CRPD) [11].

To ensure that such training leads to sustainable and measurable learning outcomes, education on disability inclusion should follow a competency-based approach. Competency-based education focuses on developing and assessing the ability of learners to apply knowledge, skills, and attitudes in real-life professional contexts [12–14]. It aims to ensure that all health professionals can demonstrate the competencies necessary to deliver equitable, person-centred, and rights-based care to persons with disabilities.

Despite increasing recognition of its importance, current health professional training demonstrates that disability-related content remains limited and fragmented [15–18]. Education and training in disability inclusion is frequently offered in the form of standalone lectures or voluntary sessions and is not accorded a high priority in terms of curriculum content. Furthermore, the provision of disability-inclusive education and training for health professionals represents a challenging endeavour in terms of sustainability. The existence of such programmes is often contingent upon the specific interests of a small number of teaching staff and are developed in a short-term manner [16, 18, 19]. Consequently, experts are calling for mandatory, longitudinal competency-based training on human rights and disability inclusion [20–23]. Such training would focus on competencies that are required to provide adequate health care for persons with disabilities and thus, contribute to achieving their right to the enjoyment of the highest attainable standard of health [15, 18–20, 24].

To develop and implement effective, mandatory and sustainable education and training on disability inclusion, it is crucial to define the specific competencies required for inclusive health care practice. Recent initiatives have been initiated to identify disability-related competencies for incorporation into health professional education and training. For instance, within the United States, the Alliance for Disability in Health Care Education has developed Core Competencies on Disability for Health Care Education relevant for all health professionals [25]. The development of competencies specifically relevant to medicine took place in India [26], while competencies most relevant to health professionals in the provision of care to persons with intellectual impairments were developed in Australia [27, 28]. Furthermore, some looked into profession- and impairment-specific aspects, such as the role and impact of specialised nurses in the care of persons with intellectual disabilities [29]. In addition to the aforementioned scientifically based standards and guidance, national and international interest groups have also developed toolkits. For instance, the Training Toolkit Inclusive Health by SeeYou Foundation [30], the Accessibility Toolkit by Ireland’s National Disability Authority [31], the Learning disability and autism programme by the National Health System (NHS) Engeland [32], or the Canadian guidelines for primary care of adults with intellectual and developmental disabilities by primary care providers [33] are just a few such examples.

Advancing competency- and human rights-based education and training for health care professionals requires clarity on which themes and content related to disability are currently represented in education and training. An overview of these themes will provide a foundation for further developing coherent, evidence-informed standards or recommendations for training and education of health professionals on disability inclusion. Therefore, the objectives of this scoping review are (1) to describe how disability-related content has been incorporated into education and training for health professionals, including the educational scope, the involvement of persons with disabilities, teaching formats, teaching methods and teaching evaluations; and (2) to identify the key themes addressed in the existing literature.

## Methods

A scoping review was conducted to identify relevant publications and evidence on training or curricula for health professionals addressing the field of disability. The scoping review approach facilitates the mapping of existing evidence and key concepts in this field, whilst simultaneously highlighting gaps in the literature and making recommendations for future research [34, 35]. A further advantage of the approach is that it allows for the inclusion of both academic and grey literature [36].

The methodological framework for scoping reviews proposed by Arksey and O’Malley [34], with subsequent modifications by Levac et al. [37] was adhered to. They include five steps: (1) Identifying the research questions, (2) Identifying relevant studies, (3) Study selection, (4) Charting the data, (5) Collating, summarizing, and reporting the results.

Reporting this scoping review was guided by the Preferred Reporting Items for Systematic Reviews and Meta-Analyses Extension for Scoping Reviews guidelines (PIRSMA-ScR) [38] (see additional file 1).

Clinical trial number: not applicable.

### Identifying the research questions

This scoping review answers the following research questions: (1) What are the main themes addressed in competency-based education and training for health professionals regarding disability, as identified in academic and grey literature? (2) How are the following characteristics reflected in existing education and trainings for health care professionals regarding disability: (2.1) Understanding of disability, (2.2) Involvement of persons with disabilities (2.3) Teaching format and (2.4) Targeted health professionals?

### Identifying relevant studies

A comprehensive literature search was conducted in the MEDLINE and Web of Science databases on 6 June 2024, encompassing all article types published since 1 January 2011, irrespective of the language of publication. The search strategy encompassed various combinations of the three core concepts: (1) education, (2) disability, and (3) health workforce (see additional file 2).

In addition to the academic literature, a series of searches were conducted on Google and Google Scholar. These searches were conducted between June 2024 and August 2024. A further publication search was conducted on the websites of Organisations for Persons with Disabilities (OPDs). A snowballing approach was adopted to capture the original research articles cited in the identified literature. This approach was applied for both, the academic and grey literature.

### Study selection

The citation management software EndNote 21.5 was used to review all identified publications for duplicates. The title and abstract screening was conducted manually by a single reviewer. The full-text screening was subsequently conducted by two researchers (JR and LG). All types of publications published in English were included - both academic literature and websites or training manuals. The target group was health care workers. In instances of uncertainty, publications directed towards professions other than health professionals, such as care workers or social workers, were incorporated if they exhibited a distinct thematic emphasis on health or health care. In the present scoping review, publications were excluded if they focused on themes other than education, training or competencies or focused solely on clinical competencies. We included publications which address disability as a such to inform human rights-based education.

### Charting the data

The research team developed a preliminary charting data form in Excel to record the content of the included studies, including training content, competencies and training evaluation. Then, the following characteristics of all included articles were charted: author(s), year of publication, type of article (primary research, secondary research, including systematic and scoping reviews, and tertiary research, such as essays and manuals), country of origin (country where study was conducted), World Health Organization (WHO) region [39] (building upon country of origin), country’s level of income (according to the classification of the World Bank) [40], geographical scale (local, subnational, national, regional, international or global), understanding of disability (medical or biopsychosocial model, human-rights based approach) [41], and impairments as differentiated in the UN CRPD, involvement of persons with disabilities, teaching format, types of impairments [3], and targeted health professionals. With respect to educational characteristics, the following characteristics were extracted by two researchers (LG and JR) from the full texts and added to an Excel spreadsheet: Educational scope (sessions, module, framework/ curriculum), involvement of persons with disabilities (includes involvement in development of training or curricula, involvement in training and teaching, involvement of family members), health professionals (mono-professional such as nurses, occupational therapists or dentists, or interprofessional; not clearly assignable), teaching format (in person or online), teaching methods, and number of persons trained when training was conducted.

The categories for the latter two characteristics were developed close to the full text. The information for the other characteristics was initially extracted as full text and then grouped into meaningful categories by the first author (LG) to reflect how key principles of disability inclusion, for example the involvement of persons with disabilities, are addressed in the trainings. The wording of the categories was selected to be closely aligned with the wording in the data material. The first draft was then reviewed and agreed upon by the team. In instances of uncertainty or disagreement regarding both, wording of category and assignment of single texts to a category, the research team engaged in deliberations to determine the most suitable category.

### Collating, summarizing, and reporting the results

Thematic analysis was conducted to identify the main themes addressed in the included publications. The themes were identified inductively-deductively capturing information presented in the included publications and guided by the human rights-based approach. More concretely, this implied that themes were extracted close to the data material (inductive coding), while at the same time being informed by key elements of a human rights-based approach (deductive coding), such as the involvement of persons with disabilities. The generation of codes, as well as the searching for and reviewing of themes, was conducted in the qualitative analysis software MAXQDA [42]. To ascertain the results pertaining to the second research question, a descriptive numerical analysis was conducted. A critical appraisal of the quality of the included publications was not performed, as such an exercise is not recommended for scoping reviews [34, 43]. Furthermore, a critical appraisal of individual sources of evidence was deemed non-pertinent as it would not contribute to the objectives of this scoping review, as described above [38].

## Results

### Overview

Of 4,164 records identified in the database search and 47 in other sources, we identified 172 records, including academic and grey literature, after full-text screening to be included in this review (Fig. 1, see Additional file 3). All publications included were available in English and are presented in alphabetical order.

### Characteristics of the included reports

The 172 included publications were assigned to primary (70%), secondary (10%), and tertiary (20%) research. The publications originate from all world regions: Region of the Americas (AMR; 30%), European Region (EUR; 17%), Western Pacific Region (WPR; 14%), African Region (AFR; 9%), Eastern Mediterranean Region (EMR; 2%), Sout-East Asian Region (SEAR; 2%), 4 publications encompassed two or three regions (4%). For 23% of the publications, the region was not applicable, e.g. for systematic reviews. In terms of geographical scale, 38% originated locally, 23% nationally, and 13% subnationally. 15% of publications were global in scope, only 1% regional or a combination of national and regional, respectively. 8% did not specify the geographical scale, or it was not applicable for the type of publication. Table 1 (see Additional file 4) provides a detailed overview of the characteristics of all included publications.

In response to objective 1 of this review, 22% of studies reported on small sessions or modules respectively, 20% on curriculum or frameworks, and for 37% of included publications no information on the educational scope was provided or it was not applicable. Out of the publications that reported on either a small session, module or curriculum/ framework, 81%, 87% and 18% respectively either implemented or recommended relevant teaching methods. These educational characteristics of the included publications are shown in Table 2 (see Additional file 5). 

### Themes identified in included publications

In response to objective 2 of this review, 19 distinct themes were identified. Table 3 (see Additional file 6) provides an overview of the themes addressed in each publication without any indication on frequency or depth of discussion within individual papers. The 19 themes are discussed in greater detail in the following sections by organising them into five groups: four themes are presented that are overarching, three themes pertain specifically to persons with disabilities, two focus on health professionals, seven themes relate to health care practice, and three concern health services and systems.

#### Overarching themes

Four themes were identified as foundational for understanding disability in general: conceptual understanding of disability, health equity, discrimination and violence, and advocacy.

##### Conceptual understanding of disability

Numerous publications emphasized that incorporating a general conceptual understanding of disability within the training curriculum is of paramount importance (12 publications) [15, 20, 25, 44–52]. A range of models of disability were used (12 publications) [23, 26, 44, 48, 53–60], including the medical model of disability (3 publications) [26, 44, 61], the social and biopsychosocial model of disability (9 publications) [18, 19, 24, 26, 49, 54, 62–64], as well as the human-rights based approach (10 publications) .

##### Health equity

A few publications have identified health equity as a central component within health professional training, delineating persons with disabilities as both vulnerable and marginalised (6 publications) [26, 46, 65–68]. Several drew comparisons between the health care experiences of persons with and those without disabilities, situating these inequities within broader institutional and social contexts (2 publications) [69, 70]. The social determinants of health, which include but are not limited to gender, ethnicity and housing, have been identified as important contributors to health inequities. Health professionals need to be enabled to more effectively recognise social determinants and their association with inequities. By doing so, they can contribute to reducing inequities in health care access and outcomes (36 publications) [15, 21, 25, 27, 44, 48, 49, 52, 54, 55, 57, 59–61, 65, 66, 69–95].

##### Discrimination and violence

Discrimination and violence were identified as salient theme in numerous publications, including raising awareness about ableism (8 publications) [76, 77, 80, 83, 95–98], its relationship to attitudes, health and health inequities (1 publication) [68], the right of non-discrimination (1 publication) [84], as well as violence and its different forms (6 publications) [25, 48, 62, 65, 89, 98]. Existing trainings addressed differences in power between persons with disabilities and those responsible for their care (5 publications) [49, 61, 65, 76, 77], enhancing sensitivity to potential signs of violence (4 publications) [62, 65, 98, 99], as well as strategies for countering discrimination (6 publications) [74, 76, 80, 95, 100, 101].

##### Advocacy

This theme included general aspects on advocacy (7 publications) [52, 57, 62, 66, 71, 96, 102], and more specifically health professionals supporting persons with disability to advocate for themselves (3 publications) [54, 62, 71], advocating for the rights persons with disabilities (6 publications) [52, 56, 62, 77, 103, 104], as well as advocating for change in the community (12 publications) [25, 30, 48, 62, 74, 82, 86, 95, 99, 105–107].

#### Persons with disabilities

Attending to and respecting the perspectives of persons with disabilities, their lifespan, as well as types of impairments and opportunities are key themes that address specifically persons with disabilities.

##### Perspectives of persons with disabilities

Focusing on the perspectives of persons with disabilities raises awareness, enhances understanding and respects values, attitudes and lived experience of persons with disabilities, especially their experiences with health care (10 publications) [27, 44, 53, 90, 92, 104, 108–111], and constitutes an opportunity for learners to develop empathy (1 publication) [109]. Relevant concepts in this context are disability culture, disability consciousness and disability humility (2 publications) [75, 112].

##### Lifespan

This theme includes the importance of biographical transitions over the lifespan – from childhood to the end of life – for qualitative care (4 publications) [20, 25, 54, 86], as well as the changing and ongoing needs of the individual throughout the life, as well as the local services that are available (5 publications) [26, 54, 71, 77, 86], and the planning and management of long-term care, including palliative care (3 publications) [113–115],

##### Types of impairments

Knowing about disability in general, different types of impairments, their causes and symptoms, underlying as well as associated health problems (15 publications) [27, 44, 46, 48, 54, 57, 66, 67, 80, 109, 116–120] was considered foundational not only for the entire care process, but also for ensuring access to health care (6 publications) [24, 86, 96, 121–123].

#### The health professional

##### Communication

Effective communication with persons with disabilities requires recognizing its central importance (23 publications) [15, 24, 25, 49, 67, 90, 102, 106, 108, 109, 115–117, 124–133], removing barriers (4 publications) [70, 83, 134, 135], and the availability of targeted trainings (29 publications) [24–27, 30, 31, 44, 53, 54, 59, 62, 71, 77, 78, 86–88, 92, 93, 99, 101, 112, 117, 118, 121–123, 126, 127, 134–147]. Such trainings include the use of tailored methods such as sign language (6 publications) , communication devices (4 publications) [62, 109, 110, 148], picture boards (1 publication) [44], avoiding abbreviations (1 publication) [126] and inappropriate language (1 publication) [53], and choosing the most appropriate communication methods (6 publications) [27, 44, 54, 92, 105, 141], while ensuring enough time (4 publications). Furthermore, effective communication and mutual understanding by providing and receiving feedback (29 publications) [25–27, 52, 54, 56, 62, 71, 78, 81, 87, 93, 102, 118, 121, 126, 135, 138, 139, 142, 144, 146, 147, 149–154], addressing the patient directly rather than their accompanying person (6 publications) [23, 25, 53, 54, 138, 144], learning to use a person’s first language (4 publications) [23, 27, 44, 142], and to communicate in a culturally competent manner (6 publications) [25, 62, 95, 103, 117, 135] are important as well. To enable inclusive communication, training should emphasize active listening (9 publications) [62, 93, 127, 138, 139, 142, 144, 154, 155], empathy (14 publications),[156, 157] respect (14 publications) [21, 25, 52–54, 62, 67, 77, 104, 118, 130, 139, 158–161], patience (3 publications) [53, 62, 144] and compassion (2 publications) [62, 139].

##### Personal conduct

In the publications included in this review, personal conduct of health care professionals was influenced by self-reflection and -awareness, including one’s privileges, values, beliefs, attitudes and biases, with regard to persons with disabilities, the impact of one’s beliefs, attitudes and biases on clinical practice and people’s health, and how those could be changed (43 publications) [162–164]. Professional and ethical attitudes and conduct, including showing dignity, respect and privacy to persons with disabilities, as well as taking personal responsibility were also addressed (14 publications) [52–54, 62, 66, 71, 78, 90, 128, 130, 144, 150, 154, 155]. The necessity for ongoing educational opportunities concerning disability (13 publications) [26, 54, 57, 74, 83, 86, 91, 102, 128, 165–168], in addition to self-reflexivity on the limitations of one’s knowledge and skills in relation to disability (7 publications) [27, 49, 57, 71, 83, 113, 151], and the dedication of health professionals to lifelong learning (12 publications) [21, 25, 27, 31, 49, 62, 68, 71, 93, 137, 149, 168], were elucidated.

#### Health care practice

##### Person-centredness

Person-centred care revealed as central to training on disability: it encompasses placing the person, as well as their families, closest support networks and important others at the centre (39 publications) [20, 21, 25, 27, 28, 44, 45, 49, 52–54, 61, 62, 70–72, 80, 82, 86, 92, 93, 96, 104, 110, 114, 117, 121, 122, 128, 130, 139, 149, 151, 159–161, 165, 169, 170], recognising them as experts in their lives, while collecting information about their lifestyles, life and medical histories (11 publications) [23, 25, 52, 62, 71, 80, 86, 93, 150, 153, 169]. Authors highlighted the need to train health professionals to respect a person’s autonomy (5 publications) [26, 44, 54, 69, 92], attend to the person’s lived experiences, their needs and abilities, values, interests, concerns and preferences when providing health care (32 publications) [25–27, 30, 49, 53, 57, 62, 71, 77, 86, 92, 93, 99, 102, 103, 111, 114, 127, 137, 139, 149, 150, 153, 155, 160, 161, 169, 171–174], and treat health issues holistically, rather than focusing only on the impairment itself (14 publications) [44, 53, 62, 77, 78, 92, 98, 106, 121, 139, 150, 160, 170, 173]. Health professionals should be able to provide reasonable accommodation (31 publications) [15, 23, 25–27, 31, 44, 49, 52–54, 57, 62, 67, 73, 77, 78, 81, 86, 92, 97, 114, 121, 122, 129, 143, 149, 161, 165, 172, 175] and individualised care plans for persons with disabilities (8 publications) [28, 71, 73, 86, 93, 118, 149, 161], under consideration of the personal and social environment (15 publications) [23, 25, 44, 49, 67, 70, 71, 77, 80, 85, 86, 161, 165, 174, 176]. Cultural competence is also needed to address diverse conceptualisations of disability that exist among both majority and minority groups (28 publications) [22, 25–27, 48, 49, 56, 57, 62, 67, 69–71, 77–79, 83, 86, 87, 92, 95, 103, 117, 128, 132, 135, 141, 172].

##### Decision-making

Decision-making with persons with disabilities requires training of health professionals (9 publications) [19, 25, 26, 54, 71, 86, 95, 118, 177] to provide patients with sufficient and accessible information (9 publications) [23, 59, 62, 71, 77, 86, 93, 121, 135], assess a person’s capacity to consent (3 publications) [26, 54, 135], and enable enabling them to give informed consent (4 publications) [93, 98, 132, 153]. Decision making needs to be tailored to individual needs, values and resources (11 publications) [25, 54, 59, 71, 77, 86, 92, 98, 135, 148, 149], a person’s cultural background (3 publications), and within the legal frame of reference (4 publications) [44, 62, 81, 98]. Health professionals need training in supported decision-making which legal representatives involved (6 publications) [54, 62, 71, 98, 135, 149]. Specific concepts such as balancing dignity of risk and duty to care (1 publication), or acknowledging the charitable view on disability as a barrier to shared decision-making (1 publication) [121] are noteworthy.

##### Accessible environment

The review identified both general factors relating to access to health care for persons with disabilities (9 publications) [25, 27, 54, 55, 70, 71, 96, 134, 139] and general accessibility (16 publications) [15, 30, 31, 44, 45, 48, 54, 55, 70, 71, 73, 74, 83, 86, 107, 122], but also specific factors related to the themes Communications, Person-centredness, and Cost and time management, as described in the respective sections. The physical environment was identified as an important theme for training, including physical accessibility and associated barriers (8 publications) [25, 53, 58, 59, 84, 118, 142, 178], strategies to improve physical accessibility (14 publications) [25, 27, 44, 48, 53, 59, 71–73, 83, 84, 98, 142, 149, 175], such as universal design (6 publications) [25, 27, 55, 59, 73, 74] and calmer and less cluttered waiting areas (1 publication).

##### Collaboration

Collaboration is a recurring theme in training on health care for persons with disabilities. It entails clear role awareness (15 publications) [27, 62, 71, 75, 86, 93, 96, 121, 122, 126, 151, 153, 171, 179, 180], timely seeking of support from others when limits are reached or assistance required (7 publications) [27, 62, 121, 126, 137, 149, 181], as well as effective team work within (27 publications) [25, 27, 44, 46, 49, 52, 54, 62, 77, 86, 93, 94, 98, 109, 110, 121, 123, 127, 130, 132, 137, 140, 161, 165, 166, 171, 173] and across institutions and settings (12 publications) [26, 27, 30, 52, 62, 71, 80, 86, 103, 149, 153, 161]. Health professionals need to be knowledgeable about available services (22 publications) [25, 27, 48, 59, 62, 66, 67, 71, 73, 80, 81, 86, 97, 114, 117, 124, 137, 140, 161, 166, 182, 183], professional scopes and responsibilities of all professionals involved (21 publications) [23, 27, 30, 49, 54, 59, 62, 67, 71, 75, 80, 86, 96, 118, 122, 126, 149, 151, 153, 161, 171], and collaborating with communities and informal caregivers, such as friends and family members, as well as a personal support network (29 publications) [25–27, 44, 54, 62, 67, 71, 80, 86, 93, 98, 99, 102, 106, 114, 118, 121, 122, 126, 127, 149, 155, 160, 165, 173, 175, 184, 185]. Education and training should also include referrals (15 publications) [15, 27, 30, 49, 62, 67, 71, 80, 86, 93, 99, 103, 118, 123, 132], case management (2 publications) [26, 132], care coordination (9 publications) [54, 71, 80, 86, 103, 106, 109, 165, 166], conflict management within teams (11 publications) [27, 56, 62, 86, 93, 102, 109, 121, 123, 155, 180], and leadership development (12 publications) [49, 62, 68, 80, 102, 105, 117, 128, 137, 155, 165, 185].

##### Evidence-based practice

Evidence-informed practice revealed as an important theme (9 publications) [52, 71, 72, 102, 105, 117, 153, 166, 167]. Health professionals should become aware of the principles of evidence-based practice and put that in the context of disability (5 publications) [59, 62, 80, 86, 93], including critically appraising knowledge of population data regarding persons with disabilities (15 publications) [46, 54, 55, 59, 62, 73, 80, 86, 93, 103, 141, 165, 186–188]. Some trainings included implementing evidence into practice (10 publications) [21, 54, 62, 71, 86, 93, 132, 165, 182, 189], and engaging appropriately in research involving persons with disabilities (12 publications) [27, 30, 54, 59, 71, 80, 86, 93, 128, 149, 165, 189].

##### Health education and health promotion

Authors highlighted the need to train health professionals in strategies related to health education for persons with disabilities so to support the health literacy of persons with disabilities (18 publications) [25–27, 54, 67, 71, 83, 88, 99, 101, 117, 132, 139, 152, 160, 182, 190, 191], and engage persons with disabilities in health education (5 publications) [26, 54, 67, 71, 132], as well as their families and support networks (4 publications) [26, 54, 71, 99] and the community (3 publications) [26, 67, 83]. Individualized interest- and resource-oriented strategies were mentioned (5 publications) [54, 88, 152, 160, 182].

A related theme to health education, yet distinct, was health promotion for persons with disabilities (15 publications) [23, 25–27, 54, 55, 67, 71, 82, 92, 93, 102, 117, 132, 182]. Health professionals need to be enabled to provide tailored, needs-based health promotion as part of personal care plans (4 publications) [25, 27, 92, 182] as well as to integrate persons with disabilities into existing health promotion programmes (2 publications) [55, 182].

##### Clinical assessment, diagnostics and therapy

Beyond general clinical competencies (6 publications) [28, 45, 68, 129, 170, 192], emphasis is placed on conducting assessments (50 publications) [23, 25–27, 46, 47, 51, 52, 54, 56–58, 67, 80, 81, 90, 92, 93, 97, 99, 101, 102, 104, 110, 112, 113, 116–118, 122, 127, 129–132, 146, 148–150, 152–154, 167, 172, 175, 179, 183, 185, 191, 193], such as taking a patient’s medical history or making observations and performing diagnostics like genetic testing (6 publications) [23, 26, 71, 80, 92, 102]. Training may include adapting these techniques to accommodate diverse needs (11 publications) [23, 25, 27, 44, 49, 54, 92, 97, 127, 132, 149] and ensuring early clinical evaluations (4 publications) [26, 80, 99, 185] to reduce misdiagnosis (1 publication) [25] and diagnostic overshadowing (1 publication) [54]. Education and training should also include effective communication about diagnoses to patients, their families and support network (4 publications) [114, 117, 158, 176]. Health professionals are trained to use clinical evaluations to inform therapy planning (14 publications) [25, 27, 44, 54, 80, 102, 104, 116, 127, 130, 132, 148, 149, 191] and adapting those to the needs of persons with disabilities (52 publications) [27, 44, 46, 49, 52, 54, 56, 58, 67, 80, 97, 99, 101, 102, 109–111, 113, 114, 116–118, 122, 127, 128, 131, 132, 140, 146, 148, 149, 152–155, 160, 161, 167, 169, 172, 173, 175, 176, 179, 183, 185, 191, 193–197]. Trainings include specific aspects such as long-term treatment, including medication adherence (8 publications) [27, 54, 67, 102, 117, 146, 152, 185], monitoring effects (8 publications) [27, 54, 67, 102, 117, 146, 152, 185], and educating persons with disabilities for self-management (3 publications) [93, 132, 160].

#### Health services and systems

##### Quality care and quality improvement

Some of the publications emphasized the importance of raising the awareness on the goal to achieve high quality care (8 publications) [27, 62, 71, 83, 121, 128, 152, 165]. Achieving this encompasses compliance with requirements (1 publication) [62], commitment (1 publication) [71] and self-reflection (1 publication) [121], coordinated resources and participation of persons with disabilities in service development (1 publication) [62], community-based health education (1 publication) [83] and minimizing risk of harm (1 publication) [165]. Also, training on quality improvement at the intersection with disability (9 publications) [27, 28, 67, 71, 86, 93, 149, 153, 165] was identified in the publications, which includes acting as a change agent (1 publication) [86], including with persons with disabilities, their families and support networks (1 publication) [27], collecting quality improvement data (3 publications) [71, 129, 149], using information technology (2 publications) [67, 165], and considerations of safety (8 publications) [27, 71, 82, 86, 93, 128, 153, 165]. Specific aspects to safety include reporting of service deficiencies (1 publication) [93], considerations of individual safety (1 publication) [62] and a safe environment (1 publication) [86], and the commitment to safety arrangements and policies (3 publications) [27, 62, 71].

##### Costs and time management

Management of time and costs in the context of education and training related to disability included flexible time management (1 publication) [121], planning sufficient time (3 publications) and balancing the burden of accessing and receiving health care, including time and money, for persons with disabilities (3 publications) [77, 93, 139]. Funding of health care for persons with disabilities (3 publications) [62, 72, 86] including insurances (1 publication) [62], coverage of costs of inclusion (1 publication) [30], and financing issues and potential alternatives in current systems (3 publications) [44, 77, 109] were addressed.

##### Laws and policies

Several publications highlighted the importance of knowing about rights of persons with disabilities especially in the context of health care (11 publications) [26, 30, 45, 72, 73, 83, 84, 90, 98, 130, 198], as well as about legislations in general as well as specifically to a country (8 publications) [31, 44, 47, 74, 86, 90, 134, 199], like the American Disability Act (5 publications) [31, 44, 47, 90, 199] or the Nigerians with Disability Decree (1 publication) [134]. Reference was given to the application of legislations in general (8 publications) [26, 27, 52, 54, 66, 80, 86, 153] and related to specific legislations such as confidentiality and privacy of data (6 publications) [23, 25, 27, 56, 71, 153]. In addition, policies specifically related to the health care of persons with disabilities were highlighted (9 publications) [27, 45, 57, 71, 79, 86, 117, 124, 200], including organisation-specific policies (1 publication) [31] and service-specific policies, e.g. for rehabilitation services (1 publication) [56].

## Discussion

This review identified key themes in the education and training of health professionals on disability, based on an analysis of 172 globally representative publications. The findings reveal a broad and multifaceted thematic landscape and offer a strong foundation for interpreting implications and guiding future directions.

Close to 75% of publications were local (38%), sub-national (13%) or national (23%). This predominance of locally driven initiatives suggests that disability inclusion efforts are largely context-specific. As has been argued by others [20, 23, 94], there is a need for coordinated efforts to develop globally aligned standards to enhance coherence and support the transfer of effective practices across professions, settings and countries. It is evident that such endeavours must be guided by universal, human rights-based principles. Health professionals play a pivotal role in health inequities experienced by persons with disabilities [1]. Consequently, the enhancement of their competencies regarding disability holds considerable potential in contributing to the reduction of health care inequities for this population group.

The 19 identified themes provide a strong foundation for developing competencies on disability inclusion that support equitable care in competency-based education. The alignment of these themes with existing frameworks has the potential to enhance their relevance and impact. One promising example is the WHO’s Global Competency and Outcomes Framework for Universal Health Coverage (UHC) [201]. Such alignment reinforces the UHC’s goal of ensuring equitable, safe and timely health care for all, including persons with disabilities. At the same time, the UHC framework would provide a strategic platform with which to embed and scale disability inclusion across health systems, thus reinforcing its role as a core component of quality and rights-based health care. Several themes from this review map directly to the six UHC domains, namely people-centredness, decision-making, communication, collaboration, evidence-informed practice, and personal conduct. In addition, other themes, including perspectives of persons with disabilities and lifespan, demonstrate conceptual alignment. Furthermore, themes such as discrimination and violence, as well as laws and policies, demonstrate a cross-cutting nature, spanning multiple domains.

This scoping review contributes to the identification of key themes to be incorporated into future curricula, providing a foundation for the development of standardized competency- and human rights-based training programs or curricula. Based on this review we have an evidence-base on content that matters, the next step is then to structure these themes into competencies and observable behaviors, encompassing knowledge, skills and attitudes. Once the competencies are defined, related learning objectives and teaching methods need to be defined, and adopted to regional, national and local contexts [202].

The establishment of a global competency standard on disability inclusion would provide a structured framework for policy alignment, curriculum reform, and monitoring progress under the UN CRPD for national governments and ministries. This, in turn, would support policy coherence, accountability, and quality assurance across health professions. The same is true for universities and higher education institutions accountable for curriculum implementation. It is recommended that professional associations adopt a bridging role by integrating disability-inclusive principles into their codes of conduct, practice guidelines, and continuing professional development. A universally accepted competency standard on disabilityinclusion would provide a cohesive framework to guide education, regulation, and practice, ensuring that all health professionals are equipped to deliver equitable, rights-based care for persons with disabilities worldwide.

The predominance of small sessions or modules on disability is congruent with the existing literature on disability curricula in health professions, which has identified limited evidence of sustained, longitudinal integration, while emphasising the value of such integration when it is in place [17, 68]. The term *longitudinal* is used to describe learners who engage with specific themes consistently over time, frequently across multiple courses or years. This process is known to foster the development of a more profound comprehension and the progressive integration of knowledge. The existing literature on longitudinal curricula demonstrates that, when implemented effectively, such models facilitate systematic repetition, reinforcement, scaffolding and progressive complexity of learning. They also enhance satisfaction, promote deeper learning and ensure continuity of experience [61, 203].

The themes identified in this review, in conjunction with the educational characteristics, indicate that professional identity formation should be considered to be integrated into competency-based education and training on disability inclusion [204, 205]. Such integration facilitates the internalisation of values of equity and inclusion alongside relevant knowledge, skills and attitudes. Consequently, there is a necessity for educational settings that deliberatively align competence development with reflective practice, mentorship, and longitudinal experiences in interacting with persons with disabilities. In this manner, competency-based education and training focuses not only on achieving observable performance outcomes but also on the developmental pathway for becoming a reflective and socially accountable professional who can provide equitable care to persons with disabilities.

The present literature demonstrates an absence of systematic evaluations of education and training for disability inclusion. The majority of evaluations are predicated on pre-post self-reports of knowledge, attitudes, or confidence, whilst a comparatively limited number assess behaviour change, institutional impact, or patient outcomes. It is worthy of note that the limited number of studies which have been conducted to evaluate the impact of the programme have reported positive effects on health professionals’ knowledge and attitudes when caring for persons with disabilities [17, 45, 57, 70, 72, 96, 103, 119, 147, 154, 169, 174, 175, 184, 188, 195, 199].

However, the overall evidence base remains methodologically limited. The use of validated, reliable evaluation tools is essential to generate meaningful and comparable evidence on the impact of disability inclusion education and training. Existing studies frequently utilise ad hoc or non-validated questionnaires, thereby compromising internal and external validity [17]. Even though the review’s primary focus did not concern evaluation, no commonly utilised instrument or framework could be identified across the included publications. The absence of standardisation compromises the ability to make comparisons and to impede the accumulation of a robust evidence base.

Furthermore, longitudinal evaluation designs are vital for assessing the sustainability of learning outcomes and their translation into professional behaviour. Such designs are indicative of the developmental trajectory of both competency acquisition and professional identity formation, which evolve over time through repeated, context-rich experiences. Incorporating validated measures within longitudinal follow-up studies has the potential to enhance methodological rigour and provide evidence on sustained behavioural, institutional, and systems-level change. In accordance with global competency standards, the evaluation of education and training on disability inclusion should incorporate persons with disabilities in its design and implementation. This participatory approach, consistent with the UN CRPD and its principle of “nothing about us without us” [3], enhances both the authenticity and validity of evaluation processes and outcomes. In doing so, it is possible to align competency standards, curriculum design and implementation with genuine needs and social realities. This, in turn, can engender a shift from a medical to a social and rights-based model of disability that promotes mutual learning and empowerment [206].

This scoping review has several limitations that should be considered when interpreting its findings. First, despite a comprehensive and systematic search strategy across multiple databases and grey literature sources, relevant materials may have been missed. Education and training on disability inclusion, such as internal training manuals or unpublished trainings, may not have been publicly available or indexed in the searched databases. Second, consistent with the scoping review methodology, no formal assessment of methodological quality was undertaken. While this approach enables the inclusion of a broad range of evidence, it precludes conclusions regarding the rigour or effectiveness of individual studies. Third, substantial heterogeneity was observed across the included publications in terms of terminology, scope and reporting detail. This variability limited comparability across studies and may have influenced the synthesis and interpretation of themes. Fourth, only publications available in English were included, introducing a potential language bias. Finally, although the review aimed to encompass all health professions, most studies focused on a limited number of disciplines or educational levels. Hence, the generalisability of findings across may be restricted. Despite these limitations, this review provides a comprehensive and detailed insight into existing evidence on education and training on disability inclusion and identifies critical gaps to inform future research.

## Conclusions

Persons with disabilities encounter health inequities, with health professionals playing a pivotal role in either preventing or perpetuating these inequities. The present review thus sought to identify the key themes that should be incorporated into the enhancement of the education and training of health professionals regarding disability inclusion. This review has yielded critical insights that identify the necessary steps to advance competency- and human rights-based education and training. Concerted efforts are required to establish a competency standard that is globally aligned. To ensure sustainable impact, competency-based education and training on disability inclusion must be designed longitudinally and combined with valid and reliable evaluation mechanisms.

## Supplementary Information

Below is the link to the electronic supplementary material.

Supplementary Material 1. PRISMA-ScR Checklist. This document illustrates the location of the various PRISMA-ScR Checklist items within this work.

Supplementary Material 2. Search strategy. This document shows the full electronic search strategy for MEDLINE and Web of Science.

Supplementary Material 3. Figure 1: PRISMA flow diagram. This figure indicates the selection of the publications and is based on “PRISMA2020 flow diagram for new systematic reviews which included searches of databases, registers and other sources“ [207].

Supplementary Material 4. Table 1: Characteristics of included publications. This table shows characteristics of the included publications, like year and type of publication, level of income in the country of training and focused types of impairment.

Supplementary Material 5. Table 2: Educational characteristics of included publications. This table shows characteristics of the realised, planned or envisioned trainings described in the included publications, like teaching methods and training evaluation.

Supplementary Material 6. Table 3: Overview of themes addressed in the included publications. This table shows key themes which were addressed in the trainings or the demands on future trainings in the included publications.

## Data Availability

All data supporting the findings of this study are available within the paper and tables (additional files).

## Declarations

No part of this manuscript has been presented at a scientific meeting or conference. Neither have the results been published in part or full or are currently submitted for publication considerations with another journal.

## Abbreviations

AFR: African Region
AMR: Region of the Americas
EMR: Eastern Mediterranean Region
EUR: European Region
OPDs: Organisations for Persons with Disabilities
PRISMA: Preferred Reporting Items for Systematic reviews and Meta-Analyses
PRISMA-ScR: PRISMA extension for scoping reviews
SEAR: South-East Asian Region
UHC: Universal Health Coverage
UN CRPD: United Nations of the Convention on the Rights of Persons with Disabilities
WHO: World Health Organization
WPR: Western Pacific Region
