# Supplementary material for: Education and training for health professionals on disability inclusion: a scoping review
Source: BMC Med Educ. 2026 Jun 20;26:1021. doi: 10.1186/s12909-026-09492-1 (PMC13289467; doi:10.1186/s12909-026-09492-1)
Supplement: Supplementary file 2 — Supplementary Material 2. Search strategy. This document shows the full electronic search strategy for MEDLINE and Web of Science. [file 12909_2026_9492_MOESM2_ESM.pdf]

Search strategy

Databases: Medline, Web of Science<br>Date: 06 June 2024	Search words	Limits
**#1**	Disab*	
**#2**	"health worker*" OR "health practitioner*" OR "health professional*" OR „health assistant*" OR "health workforce" OR "health personnel" OR "health staff" OR "health cadre"	
**#3**	"educat*" OR "training" OR "curricul*"	
**#4**	"competenc* framework" OR "competenc* model"	
**#5**	#3 OR #4	
**#6**	#5 AND #1 AND #2	
**#7**	#6	Publication date:<br>Starte date: 01 January 2011<br>End date: 06 June 2024
