# Supplementary material for: Education and training for health professionals on disability inclusion: a scoping review
Source: BMC Med Educ. 2026 Jun 20;26:1021. doi: 10.1186/s12909-026-09492-1 (PMC13289467; doi:10.1186/s12909-026-09492-1)
Supplement: Supplementary file 3 — Supplementary Material 3. Figure 1: PRISMA flow diagram. This figure indicates the selection of the publications and is based on “PRISMA2020 flow diagram for new systematic reviews which included searches of databases, registers and other sources“ [207]. [file 12909_2026_9492_MOESM3_ESM.pdf]

PRISMA 2020 flow diagram for new systematic reviews which included searches of databases, registers and other sources

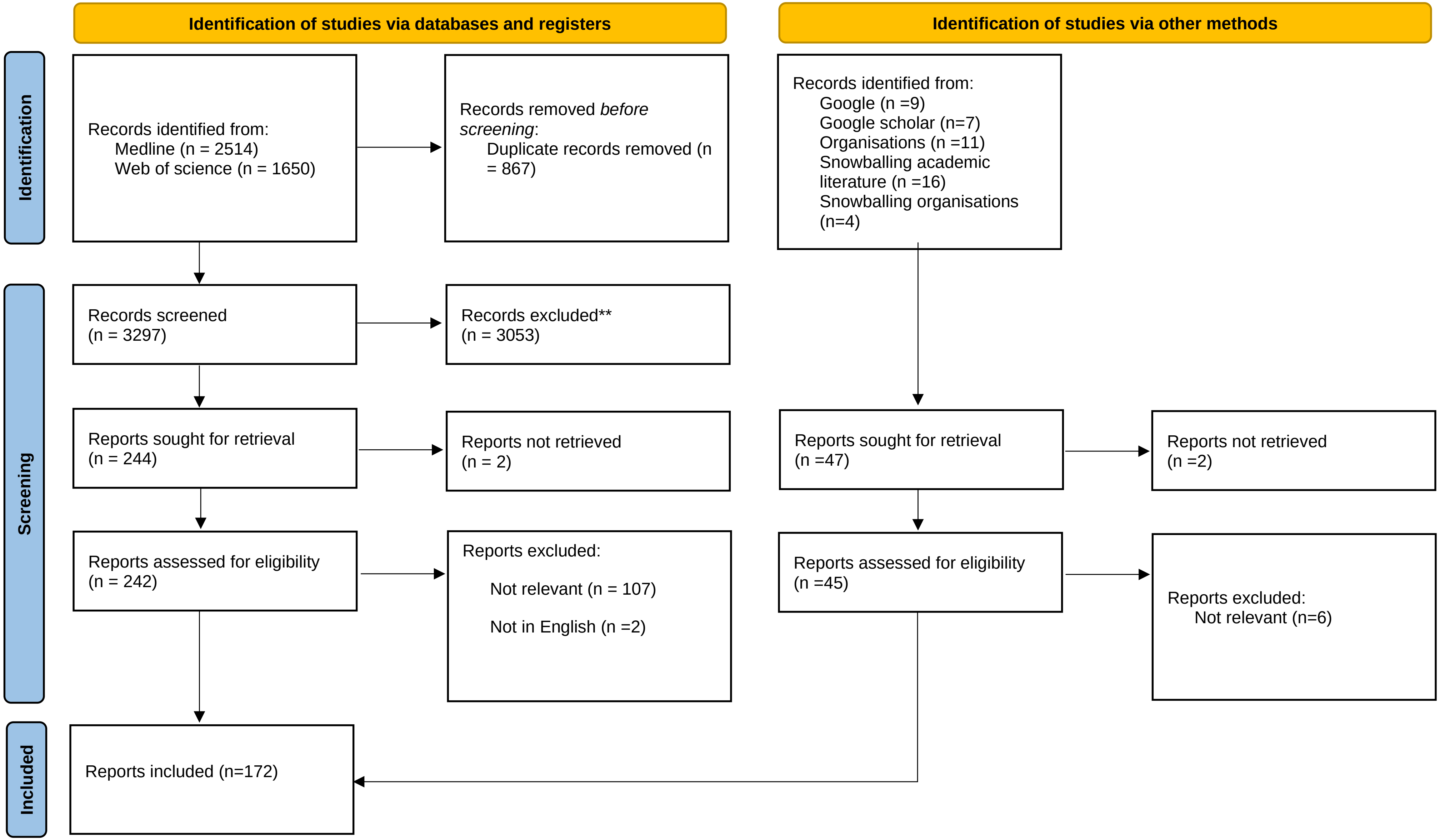

*Consider, if feasible to do so, reporting the number of records identified from each database or register searched (rather than the total number across all databases/registers).
**If automation tools were used, indicate how many records were excluded by a human and how many were excluded by automation tools.

Source: Page MJ, et al. BMJ 2021;372:n71. doi: 10.1136/bmj.n71.

This work is licensed under CC BY 4.0. To view a copy of this license, visit https://creativecommons.org/licenses/by/4.0/
